# Implant Dentistry: New Materials and Technologies 2020

**DOI:** 10.1155/2021/9852932

**Published:** 2021-11-25

**Authors:** Luigi Canullo, Henriette Lerner, Paolo Pesce

**Affiliations:** ^1^University of Valencia, Valencia, Italy; ^2^Johann Wolfgang Goethe-University, Frankfurt am Main, Germany; ^3^University of Genova, Genova, Italy

While osseointegration has become already clinically established concept in healthy patients, clinicians are facing a growing number of implant-supported rehabilitations on pathophysiologically impacted bone.

In these scenarios, moderately rough surfaces/traditional protocols might represent a *locus minoris resistenziae*. In fact, a high-performance surface might be requested for a long-standing integration of the fixture.

Bioactive surfaces with increased surface energy might respond to this requisite without encountering possible higher risk of bacterial contamination as rough-surfaced implants demonstrated.

Actually, the surface energy directly correlates with hydrophilicity and (on the contrary) indirectly with the presence of contaminants on the surface. In fact, it decreases with increased surface deposition of atmospheric elements or pollutants (present even on the “sterile” new implants).

As demonstrated, decontamination of the implant surface is an essential prerequisite for cell adhesion. However, even in optimal conditions of surface decontamination, the titanium fixture still remains hydrophobic and, then, less “tissue friendly.” This is correlated to the oxidation of the external titanium layers due to the presence of oxygen into the implant sterile package.

The bioactivation through chemical or biophysical methods increases fixture surface energy and then wettability, removing the oxidized external layers.

The biological advantage of such activation is both qualitative (higher number of adhered cells) and quantitative (flat vs. spreaded arrangement), with a stronger adhesion, and this implies a faster cell adhesion and better cell stratification.

Translated to the clinics, this strategy promises to result in stronger osseintegration even in the initial stages of the treatment in physiological quality bone or after the traditional timing in compromised bone-quality patients.

*Luigi Canullo* *Henriette Lerner* *Paolo Pesce*

